# Glial Cells and Synaptic Plasticity

**DOI:** 10.1155/2016/5042902

**Published:** 2016-05-17

**Authors:** Fushun Wang, Tifei Yuan, Alfredo Pereira, Alexei Verkhratsky, Jason H. Huang

**Affiliations:** ^1^Department of Psychology, Nanjing University of Chinese Medicine, Nanjing 210023, China; ^2^Center of Translational Neuromedicine, Department of Neurosurgery, University of Rochester, Rochester, NY 14643, USA; ^3^Department of Neurosurgery, Baylor Scott & White Health, Temple, TX 76508, USA; ^4^Department of Psychology, Nanjing Normal University, Nanjing, Jiangsu 210023, China; ^5^Institute of Bioscience of Botucatu, Sao Paulo State University, 18618-970 Botucatu, SP, Brazil; ^6^Department of Life Science, University of Manchester, Oxford Road, Manchester M139PT, UK; ^7^Department of Surgery, Texas A&M College of Medicine, Temple, TX 76504, USA

Neuroglia are composed of highly heterogeneous cellular populations of neural (astrocytes, oligodendrocytes, and NG2 glial cells) and nonneural (microglia) origin that are essential for maintaining efficient neurotransmission, homeostatic cascades, supply of energy metabolites, turnover of neurotransmitters, and establishment of the blood-brain barrier [1]. Astrocytes shape synaptic networks through essential roles in synaptogenesis, synaptic maturation, and synaptic extinction [2, 3]. Furthermore, astroglial cells secrete neurotransmitters (such as glutamate, ATP, and GABA), neuromodulators (such as adenosine and D-serine), neurohormones (such as atrial natriuretic peptide), and other humoral factors (such as eicosanoids) that modulate synaptic networks and affect information processing [4]. The concept of “multipartite synapse” formalizes the multicomponent nature of the synaptic concept that includes astroglial perisynaptic processes, microglial processes, and extracellular matrix [5, 6].

Potentiation and depression of synaptic connections are critical for learning, memory formation, and emotions [7, 8]. Long-term potentiation (LTP) and long-term depression (LTD) are triggered by patterned and repeated synaptic activities, depending on the complex dynamics of neurotransmitters (especially glutamate) in the synaptic cleft. Both the temporal course and spatial distribution of glutamate contribute to the coordinated activation of intracellular signaling cascades affecting synaptic strength [9]. The multipartite synapse concept has defined astrocyte as the key regulator of glutamate homeostasis (mediated through release and uptake) [10]. Astrocytes, for example, are capable of releasing D-serine to enhance the function of NMDA receptors [11]. In addition, astrocytes can change the buffering ability to take up extracellular K^+^, thus modulating synaptic plasticity [12]. Calcium dynamics in astrocytes determine the release of glutamate and ATP molecules. At the synaptic level, the astroglial calcium signaling is activated in response to synaptic activities, such as repeated synaptic stimulation, through purinergic, glutamatergic, and cholinergic pathways. The hyperactivity of neural circuits (e.g., in epilepsy) results in altered calcium dynamics in astrocytes. These changes, in turn, contribute to the differential modulation of synaptic efficacy under physiological or pathological circumstances [13, 14].

The papers collected in this special issue focus on glial cells and synaptic plasticity. The reviews and experimental papers present the evidence that glial cells indeed affect long-term synaptic changes.

In “Housing Complexity Alters GFAP-Immunoreactive Astrocyte Morphology in the Rat Dentate Gyrus,” G. Salois and J. S. Smith [15] demonstrate that the housing environment can affect neural plasticity. They found that an enriched environment results in considerable neuroplasticity in the rodent brain. They used confocal microscopy and found that astrocytes play a key role in the process and induce changes in synaptic spines. These findings offer a hallmark feature for the understanding of numerous diseases, including the neurodegenerative ones.

In “Recent Advance in the Relationship between Excitatory Amino Acid Transporters and Parkinson's Disease,” Y. Zhang et al. [16] reviewed their studies and also recent discoveries about the excitatory amino acid transporters (EAATs). Glutamate is the major excitatory neurotransmitter in the central nervous system, and it is mostly removed by astrocytes, where it is converted into glutamine. Impairment of astroglial glutamate uptake leads to the accumulation of glutamate in the synaptic cleft, which may contribute to various pathologies such as Parkinson's disease (PD).

Consistent with recent studies about astrocytic function in emotions, the paper “Anger Emotional Stress Influences VEGF/VEGFR2 and Its Induced PI3K/AKT/mTOR Signaling Pathway,” by P. Sun et al. [17], reported changes of VEGR/VEGFR2 in both astrocytes and neurons, induced by the anger emotion; these changes, in turn, can stimulate neurogenesis.

In the review article “The Plastic Glial-Synaptic Dynamics within the Neuropil: A Self-Organizing System Composed of Polyelectrolytes in Phase Transition,” V. M. F. de Lima and A. Pereira Jr. [18] reported another pathway for neuronal-glial interaction: the plastic nonlinear dynamics between glial and synaptic terminals; they also offered a model based on hydroionic waves within the neuropil.

In the paper “Glia and TRPM2 Channels in Plasticity of Central Nervous System and Alzheimer's Diseases,” J. Wang et al. [19] review recent findings about synaptic plasticity in neurodegenerative diseases, mainly focusing on the transient receptor potential melastatin 2 (TRPM2) channels. The TRPM2 is a nonselective Ca^2+^ permeable channel expressed in both glial cells and neurons, which regulates synaptic plasticity and also the glial cells. In this review, authors summarized recent discoveries about the contribution of TRPM2 in physiological and pathological conditions.

In “Dynamic Alterations of miR-34c Expression in the Hypothalamus of Male Rats after Early Adolescent Traumatic Stress,” C. Li et al. [20] reported experimental findings about neural plasticity under stress. They found that stress induces the overexpression of several types of microRNA notably including corticotrophin releasing factor 1 (CRFR1 mRNA) and miR-34c. Expression levels of the miR-34c in the hypothalamus represent an important factor involved in susceptibility to posttraumatic stress disorders.

In the subsequent paper “Role of MicroRNA in Governing Synaptic Plasticity,” Y. Ye et al. reviewed the role of microRNA in neural plasticity. They explored recent findings demonstrating that miRNA exerts widespread regulation over the translation and degradation of target genes in the nervous systems and contributes to the pathophysiology of plasticity-related diseases.

In “Astrocyte Hypertrophy Contributes to Aberrant Neurogenesis after Traumatic Brain Injury,” C. Robinson et al. reported their recent findings about astrocytic changes after traumatic brain injury (TBI). They analyzed the immunohistochemistry of glial fibrillary acidic protein and doublecortin and found a loss of radial glial-like processes extending through the granule cell layer after TBI. They further suggested that hypertrophied astrocytic processes form an ectopic glial scaffold that might facilitate the aberrant development of immature neurons in the dentate gyrus.

In “Modulation of Synaptic Plasticity by Glutamatergic Gliotransmission: A Modeling Study,” M. De Pittà and N. Brunel reported a computational model about gliotransmitter releasing pathways related to modulation of synaptic release and postsynaptic slow inward currents. This model predicts that both pathways could profoundly affect synaptic plasticity.

Collectively, these studies demonstrate that glial cells play an important role in neural plasticity under physiological and pathological conditions. We hope that this special issue will stimulate interest in the field of glial cells modulating synaptic activities and will help to achieve a deeper understanding of the role of glial cells in neural plasticity.
